# A Tunable Terahertz Absorber Based on Double-Layer Patterned Graphene Metamaterials

**DOI:** 10.3390/ma16114166

**Published:** 2023-06-02

**Authors:** Xin Tang, Haoduo Jia, Luyang Liu, Ming Li, Dai Wu, Kui Zhou, Peng Li, Langyu Tian, Dingyu Yang, Weijun Wang

**Affiliations:** 1 Institute of Applied Electronics, China Academy of Engineering Physics, Mianyang 621000, China; townsonme@outlook.com (X.T.);; 2 College of Optoelectronic Engineering, Chengdu University of Information Technology, Chengdu 610225, China; 3 School of Hydraulic and Ecological Engineering, Nanchang Institute of Technology, Nanchang 330099, China

**Keywords:** terahertz, metamaterial, absorber, graphene

## Abstract

Graphene is widely used in tunable photonic devices due to its numerous exotic and exceptional properties that are not found in conventional materials, such as high electron mobility, ultra-thin width, ease of integration and good tunability. In this paper, we propose a terahertz metamaterial absorber that is based on patterned graphene, which consists of stacked graphene disk layers, open ring graphene pattern layers and metal bottom layers, all separated by insulating dielectric layers. Simulation results showed that the designed absorber achieved almost perfect broadband absorption at 0.53–1.50 THz and exhibited polarization-insensitive and angle-insensitive characteristics. In addition, the absorption characteristics of the absorber can be adjusted by changing the Fermi energy of graphene and the geometrical parameters of the structure. The above results indicate that the designed absorber can be applied to photodetectors, photosensors and optoelectronic devices.

## 1. Introduction

With the advancement of terahertz technology, terahertz metamaterial absorbers exhibit significant application values and development prospects in fields such as sensing, imaging, filtering, electromagnetics, absorption, detection and so on [1,2,3,4,5]. Traditional metamaterial absorbers are usually designed and fabricated with metal materials such as gold, silver and aluminum. Typically, they consist of a multilayer stack [6] comprising a metal pattern layer on the top, an insulated dielectric layer in the middle and a metal substrate layer at the bottom. Since the perfect absorber was first proposed by Landy, it has become a widely studied field. Researchers have designed models of metamaterial absorbers in a variety of structures and investigated their properties in single, multi-band and broadband absorption [7,8,9,10]. However, once absorbers designed with metal materials have been fabricated, their optical application characteristics are permanently fixed. This means that parameters such as resonant frequency and amplitude will be fixed and unable to be actively adjusted. To change such parameters, the structure of the absorber must be redesigned and manufactured again, which cannot meet the needs of flexible absorption.

To address the above challenges, researchers in this field turned to graphene [11], a remarkable two-dimensional material with carbon atoms arranged in a regular manner that exhibits excellent optical, electrical and mechanical properties [12]. In the terahertz and mid-infrared frequency ranges, graphene can support plasmon resonance [13,14,15,16]. Compared to metals, graphene plasmon has the advantages of low loss, strong slow-wave effect and dynamic tunability [17,18,19,20], which makes it highly valuable for optical conversion, metamaterials, nonlinear optics and other fields. Researchers have attempted to substitute metals with graphene to achieve absorption of the incident waves. Utilizing its dynamic tunability, researchers have designed high-performance metamaterial absorbers [21,22,23,24]. Furthermore, due to the strong interaction between graphene and terahertz, the designed absorber can also be applied in various fields.

In this paper, we propose a broadband absorber that is polarization independent and angle insensitive. It is composed of disc graphene, open ring graphene, two insulating dielectric layers and a metal base substrate. According to the simulation results, it achieves over 90% absorption in the 0.53–1.50 THz range. Within the range of 0.59–1.38 THz, the absorption rate exceeds 95%. The mechanism of perfect absorption is revealed through the analysis of the electric field and current distribution diagrams. The effect of different incidence conditions is also explored, and the results reveal that the designed absorber is insensitive to polarization and angles. The absorption can also be enhanced efficiently by adjusting the Fermi energy and dimension of graphene. In addition, a comparison with published graphene absorbers has been conducted to provide valuable insights for the design and optimization of future absorbers.

## 2. Method

**Figure 1:**
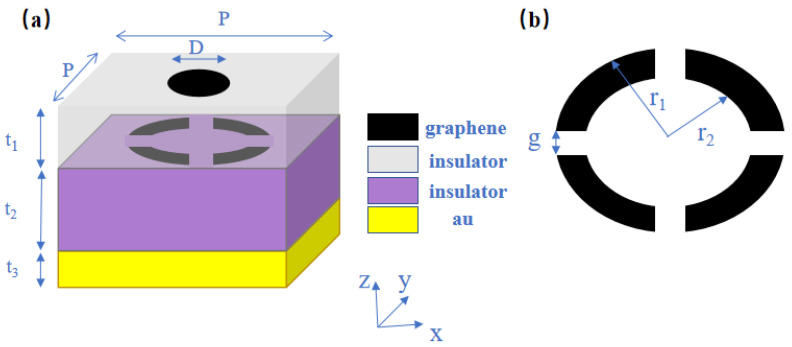
(**a**) Schematic diagram of the absorber structural unit. (**b**) Schematic diagram of the open ring graphene layer.

The schematic diagram and geometric parameters of the double-layer graphene absorber proposed in this paper are shown in Figure 1. The absorber unit structure was periodically arranged in the x-direction and the y-direction and the period was P=100 μm. The structure consisted of five parts; from top to bottom, there is a disk graphene layer with a diameter of  D=10 μm, an insulating dielectric layer with a thickness of $t_{1}=10\;\mu m$, an open ring graphene layer (Figure 1b), a polyimide (PI) layer with a thickness of $t_{2}=40\;\mu m$ and a metal bottom layer with $t_{3}=200\;\mathrm{nm}$. The thickness of the metal bottom layer greatly surpasses the skin depth of the incident wave, blocking the transmission of the incident wave [25]. Assuming that the insulating layer is lossless, its dielectric constant is 1.96 [26]. For the top layer open ring graphene, the outer radius of the ring is $r_{1}=50\;\mu m$, the inner radius is $r_{2}=30\;\mu m$ and the opening width between the rings is g=4 μm.

For the top layer of graphene, a disc shape was deliberately selected to provide uniform properties when external waves come in from every direction, guaranteeing uniformity within the interior of the structure. For the middle layer of graphene, the open ring shape was chosen to better generate resonance and accomplish perfect absorption. The scheme of applying multiple graphene layers alternating with dielectric layers can better bind the electromagnetic field energy of the incident waves within the structure.

In this study, graphene is regarded as a conductive surface. The thickness of graphene $t_{g}=1\;\mathrm{nm}$ [27], and the surface conductivity of graphene is composed of the intraband and interband conductivity. The surface conductivity of graphene can be expressed by the Kubo formula as [3,28]:

(1) $$\sigma_{w}=\frac{2e^{2}k_{B}T}{\pi \hbar^{2}}\frac{i}{\omega +{i\tau }^{-1}}\ln\left[2\cos h\left(\frac{E_{F}}{2k_{B}T}\right)\right]+\frac{e^{2}}{4\hbar^{2}}\left[\frac{1}{2}+\frac{1}{\pi }\arctan\left(\frac{\hbar \omega -2E_{F}}{2k_{B}T}\right)\right]\;-\frac{e^{2}}{4\hbar^{2}}\left[\frac{i}{2\pi }\ln\frac{{\left(\hbar \omega +2E_{F}\right)}^{2}}{{\left(\hbar \omega -2E_{F}\right)}^{2}+4{\left(k_{B}T\right)}^{2}}\right]$$

In the formula, e is the electron charge, $k_{B}$ is the Boltzmann constant, T=300 K is the temperature, ω is the angular frequency of the incident wave, $\tau ={\mu E}_{F}\left({\mathrm{eV}}_{F}\right)$ is the relaxation lifetime of the carrier, $\hbar =\frac{h}{2\pi }\;$ is the Planck constant, $E_{F}$ is the Fermi energy, the Fermi velocity is $V_{F}=1\times 10^{6}\;m\cdot s^{-1}$ and the current carrier mobility is $\mu_{c}=10,000{\;\mathrm{cm}}^{2}\cdot V^{-1}\cdot s^{-1}$ [29]. The graphene conductivity is related to factors such as incident wave frequency, temperature, Fermi energy and carrier relaxation lifetime. The carrier concentration of graphene determines the Fermi energy, while the carrier concentration can be continuously adjusted by chemical doping or applying bias voltage. In the terahertz frequency, when the photon energy $\hbar \omega \ll E_{F}$, the surface conductivity of graphene is dominated by the intraband. According to the Pauli exclusion principle, the interband conductivity can be ignored, and its surface conductivity can be approximately expressed as [29,30]:

(2) $$\sigma_{w}=\frac{e^{2}E_{F}}{\pi \hbar^{2}}\frac{i}{\omega +{i\tau }^{-1}}$$

In this study, the absorptivity A can be expressed as A=1−R−T, where R and T are the reflectance and transmittance, respectively. The thickness of the designed bottom metal surpassed the skin depth of the incident wave, which was equivalent to blocking the transmission of the incident wave, making the transmittance T=0. Therefore, the absorptivity can be simplified as A=1−R.

## 3. Results and Discussions

**Figure 2:**
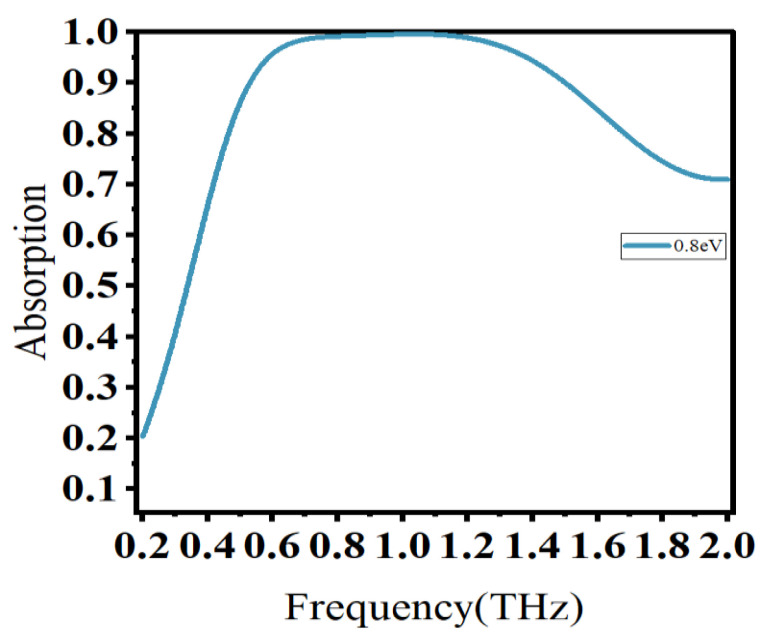
Absorption characteristic curve.

We used CST Microwave Studio 2019, commercial software for numerically simulating high-frequency electromagnetic fields. Specifically, we adopted the frequency domain solver module to set the structure with periodic boundary conditions in the x and y directions and open boundary conditions in the z direction. The incident wave was vertically incident from the upper surface of the absorber, and the entire computational field was discretized using a user-controlled tetrahedral grid. The Fermi energy of graphene is $E_{F}\;$= 0.8 eV and the absorption characteristic curve can be observed in Figure 2.

As illustrated in Figure 2, the absorption characteristic curve of the absorber exhibited a broadband absorption peak. Different from the single resonance peak generated by conventional absorbers, it was formed by multiple resonance lines superimposed on each other. It ranged from 0.53 THz to 1.50 THz, with absorption exceeding 90%, surpassing the traditional metal or single layer broadband absorbers. Moreover, within the range of 0.59–1.38 THz, the absorption exceeded 95%, indicating the perfect absorption of incident waves at specific frequencies.

**Figure 3:**
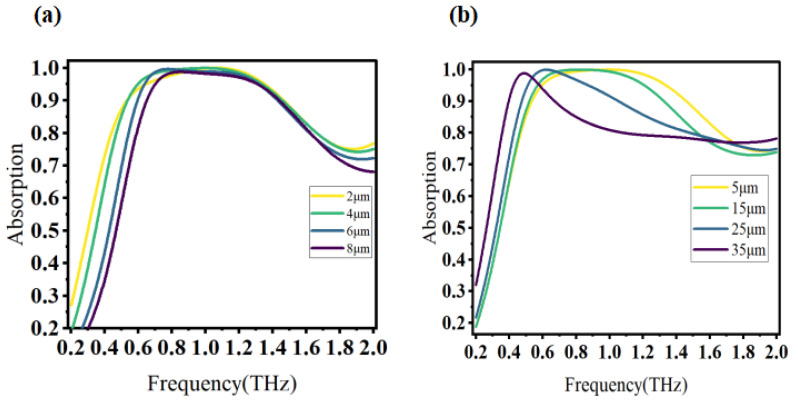
(**a**) The absorptivity curve of the open ring with different widths. (**b**) The absorptivity curve of the disc with different radii.

To investigate the influence of the double-layer patterned graphene layer on absorption characteristics, we maintained other material and structural parameters unchanged and modified the opening width of the open ring graphene layer and the radius of the disk graphene, respectively. The Fermi energy of graphene was set to 0.8 eV, and simulation frequency ranged from 0.2 THz to 2 THz. The resulting changes are illustrated in Figure 3.

The width of the open ring graphene was adjusted to 2 μm, 4 μm, 6 μm and 8 μm, as shown in Figure 3a. With the increase in the opening width, the center frequency shifted to a high frequency, indicating a blue shift trend. However, the absorption bandwidth and absorption rate both decreased after 4 μm, indicating that the impedance match was achieved at 4 μm. Figure 3b illustrates the effect of modifying the graphene disk radius. With the increase in the disk radius, the center frequency gradually shifted to lower frequencies, reflecting a red shift trend, while the absorption bandwidth steadily decreased. Based on the influence of the above changes on the absorption performance, we chose the optimal structural parameters as an opening width of 4 μm and a disk radius of 5 μm. 

The above phenomenon can be well explained using impedance matching theory. The absorber is regarded as an integral structure, and as previously mentioned A=1−R; it is necessary to minimize the reflectivity R to achieve the best absorption performance. Reflectivity can be expressed as $R={\left|S_{11}\right|}^{2}$, and it is determined by the reflection coefficient $S_{11}$. The magnitude of the reflection coefficient depends on the matching degree between the equivalent impedance of the absorber and the impedance of free space. The reflection coefficient $S_{11}$ can be expressed as follows:

(3) $$S_{11}=\frac{Z_{1}-Z_{0}}{Z_{1}+Z_{0}}$$

where $Z_{1}$ is the equivalent impedance of absorber, and $Z_{0}$ is the impedance of free space. When the equivalent impedance of absorber perfectly matches the free space impedance, it can be concluded that $Z_{1}=Z_{0}$. This means that the absorption performance is optimal at this point. Figure 3 shows that adjusting the structural parameters leads to changes in the equivalent impedance of absorber $Z_{1}$.

**Figure 4:**
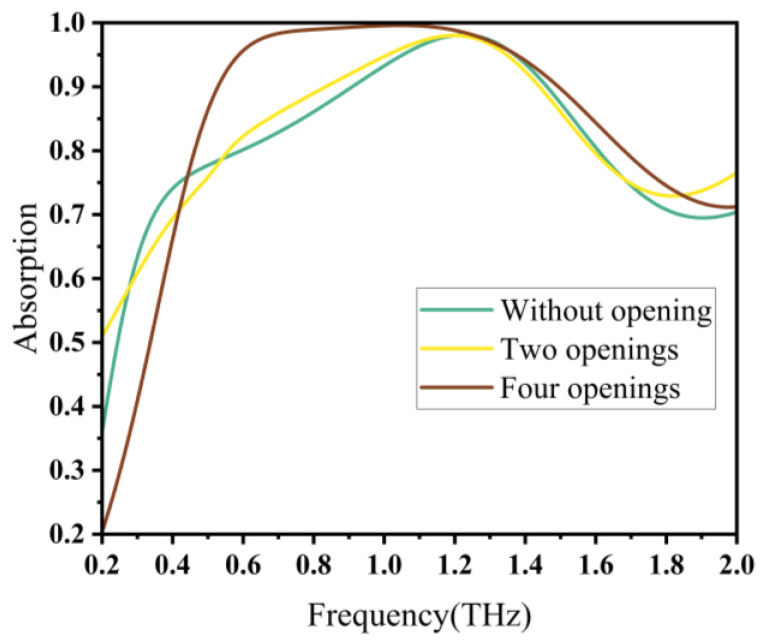
Absorption characteristics at different opening times.

Furthermore, we investigated the number of openings in the ring structure, as shown in Figure 4. When the ring was not closed, the desired resonance failed to generate and perfect bandwidth absorption was not achieved. When the ring was opened twice, the equivalent impedance of absorber was not perfectly matched to the impedance of free space, resulting in a low absorption rate and a narrow bandwidth. However, when the ring was opened four times, the overall structure exhibited a high symmetry and the impedance was perfectly matched. The overall absorption rate and absorption bandwidth were optimal at this point.

**Figure 5:**
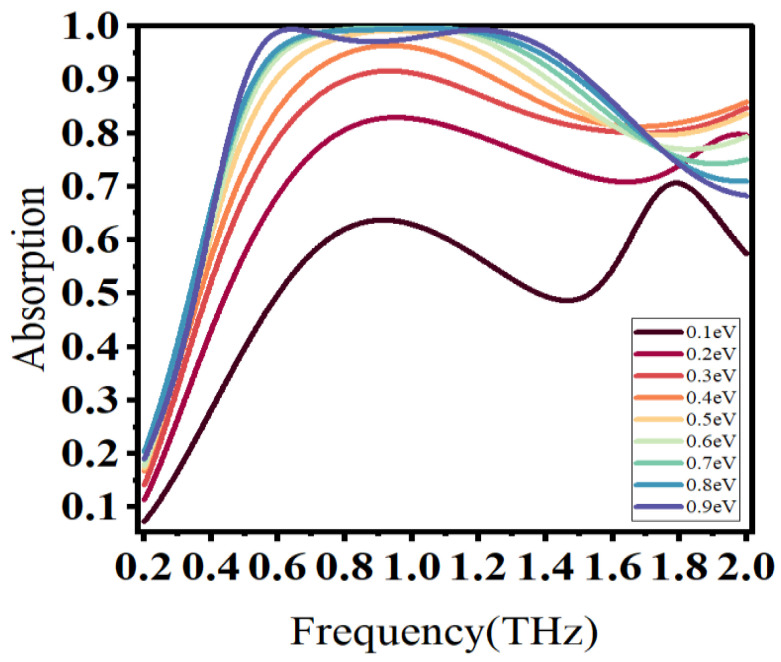
Absorption characteristics at different Fermi energy levels.

To explore the dynamic tunable properties of the designed absorber, we changed the Fermi energy of graphene $E_{F}$. The corresponding absorption characteristics are shown in Figure 5. As the Fermi energy increased, the resonance frequency exhibited a blue shift phenomenon and the resonance absorption peak gradually increased. This is because the increase in Fermi energy enhances the dielectric constant of graphene and consequently enhances the electromagnetic resonance effect of the absorber. However, when the Fermi energy exceeds 0.8 eV, the absorption peak tends to decrease. This implies that optimal absorption occurs at 0.8 eV. Dynamic tunability enhances the application potential of the absorber.

**Figure 6:**
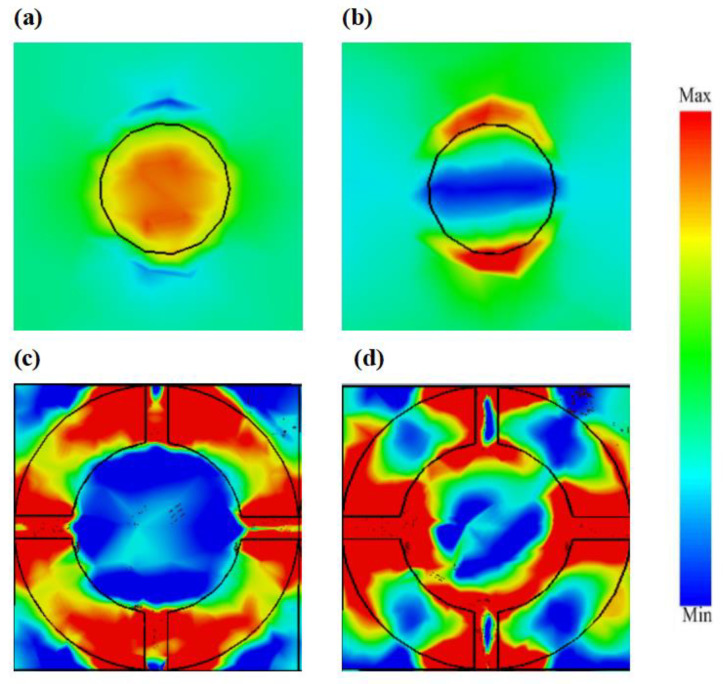
Electric field distribution of waves linearly polarized along the OY axis. (**a**) Disc graphene $E_{F}=0.1\;\mathrm{eV}$. (**b**) Disc graphene $E_{F}=0.8\;\mathrm{eV}$. (**c**) Open ring graphene $E_{F}=0.1\;\mathrm{eV}$. (**d**) Open ring graphene $E_{F}=0.8\;\mathrm{eV}$.

**Figure 7:**
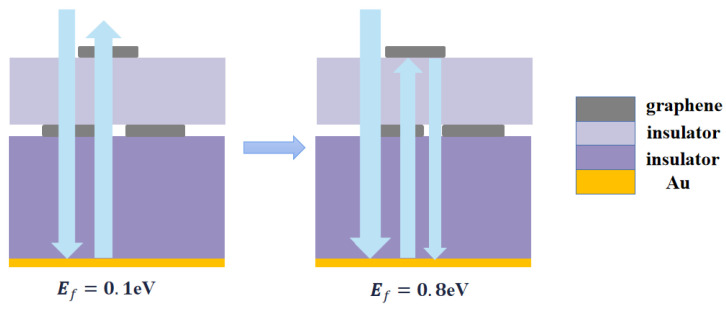
Schematic diagram of absorption at different Fermi energy levels.

To better investigate the absorption mechanism of the proposed absorber, we also simulated the electric field distribution of incident waves. Figure 6 shows the electric field distribution of waves linearly polarized along the OY axis at the Fermi energy of 0.1 eV and 0.8 eV. To illustrate the electric field distribution clearly, we introduced the multiple interference theory for analysis. When the Fermi energy was 0.1 eV, the incident electromagnetic waves were reflected after reaching the metal bottom surface, with a portion resonating on the open ring graphene. This indicates that the absorption is not ideal at this point. When the Fermi energy increased to 0.8 eV, a significant amount of incident electromagnetic waves entered the absorber, eventually generating a resonant cavity within the structure. The electromagnetic waves repeatedly reflected in the resonant cavity and the interference between incident and reflected waves led to the absorption of waves. As a result, the perfect absorption of incident waves was achieved. The schematic diagram outlining this phenomenon is displayed in Figure 7.

When the Fermi energy level surpassed 0.8, the top disc graphene exhibited metal-like properties. As a result, when significant amounts of incident electromagnetic waves passed through the top graphene, part of them were reflected, which led to fewer electromagnetic waves entering the interior of the structure. Such a reduction inevitably causes a decrease in the absorption performance. Consequently, when designing graphene absorbers, it is not always optimal to increase the Fermi energy.

From our study, it can be concluded that two key factors need to be optimized for perfect absorption. First, it is crucial to ensure that the incident electromagnetic waves are maximized into the interior of the structure. Second, it is necessary to adjust the structural parameters for generating stronger resonances and enhancing the absorption of incident electromagnetic waves within the structure. The above findings provide informative inspiration for the future of absorbers design.

**Figure 8:**
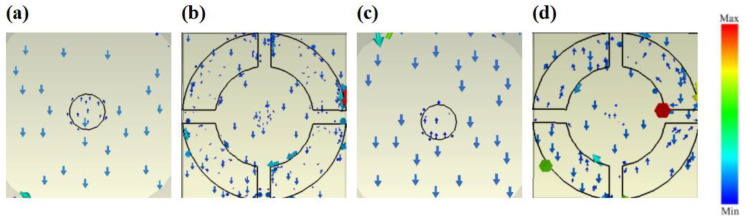
Current distribution of waves linearly polarized along the OY axis. (**a**) Disc graphene $E_{F}=0.1\;\mathrm{eV}$. (**b**) Open ring graphene $E_{F}=0.1\;\mathrm{eV}$. (**c**) Disc graphene $E_{F}=0.8\;\mathrm{eV}$. (**d**) Open ring graphene $E_{F}=0.8\;\mathrm{eV}$.

Figure 8 demonstrates the current distribution of waves linearly polarized along the OY axis. Both disk graphene and open ring graphene exhibit current arrow directions that undergo back-and-forth oscillations in a single direction, indicating the dipole resonance excited by the incident wave. Hence, the designed absorber’s resonance mode is the dipole resonance mode.

**Figure 9:**
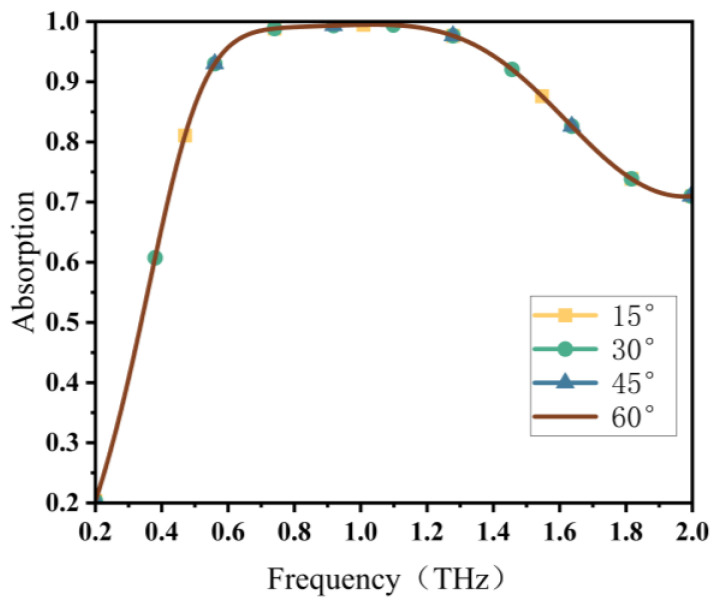
Absorption characteristics at different polarization angles.

In practical applications, a critical factor to be considered is the polarization sensitivity of the absorber. As depicted in Figure 9, with the polarization angles set at 15°, 30°, 45° and 60°, their corresponding absorption curves perfectly overlapping was observed. This was due to the high symmetry of the absorber, and changing the polarization angles has no influence on the absorption performance. As a result, the designed absorber is polarization insensitive.

**Figure 10:**
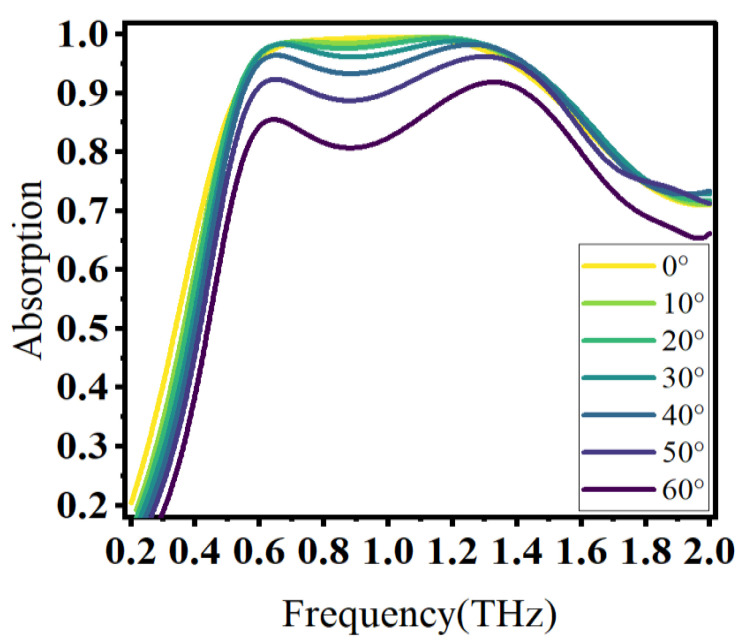
Absorption characteristics at different incident angles.

The above discussion is based on the case where the electromagnetic wave is incident vertically. In practical applications, it is difficult to incident vertically all the time. To verify that the designed absorber had the absorption performance of wide angle incidence, the angle of inclined incidence, angle θ, was defined as the angle between the wave vector and the normal. The incident angle increased from 0° to 60°, as shown in Figure 10.

As the angle of incidence increased gradually in the range of 0° to 50°, the absorption performance showed outstanding performance. Once the angle of incidence increased to 60°, it could be observed that the absorption performance decreased. The potential reason was the reduction in the electric field component parallel to the absorber surface, which led to a weaker interaction between the incident wave and the graphene. Nevertheless, the overall absorption remained above 80%. In conclusion, the proposed absorber effectively demonstrated a remarkable insensitivity to wide incidence angles.

In our comparison with previously published graphene absorbers, we evaluated the three crucial performance criteria of broadband absorption, incidence angle insensitivity and polarization angle insensitivity. While most previous studies excelled in only one of the three criteria, our designed absorber exhibits a promising performance in all three areas. This critical observation indicates that the designed absorber is more versatile and adaptable for wider applications and scenarios. As a result, our absorber provides a valuable reference for future absorbers’ design.

## 4. Conclusions

In summary, this paper proposes a novel idea for designing wide-angle incidence insensitive absorbers through the utilization of double-layer patterned graphene. Simulation results demonstrated that this absorber achieved exceptional absorption performance independent of polarization angles. Additionally, the study highlights that the absorber’s performance could be significantly improved by adjusting both the Fermi energy and geometric parameters. Consequently, the designed absorber is remarkable as it is characterized by dynamic tunability, multilayer structural stacking and broadband absorption capability, making it a valuable reference for the design and fabrication of future absorbers.

## Data Availability

The data that support the findings of this study are available from the corresponding author upon reasonable request.
